# The risk of hyperuricemia assessed by estimated glucose disposal rate

**DOI:** 10.3389/fendo.2025.1567789

**Published:** 2025-06-18

**Authors:** Zhaoxiang Wang, Ruoshuang Liu, Fengyan Tang, Yirong Shen

**Affiliations:** ^1^ Department of Endocrinology, Affiliated Kunshan Hospital of Jiangsu University, Kunshan, Jiangsu, China; ^2^ Department of Endocrinology, The First Affiliated Hospital of Nanjing Medical University, Nanjing, Jiangsu, China; ^3^ Department of Clinical Nutrition, Hangzhou Hospital of Traditional Chinese Medicine, Hangzhou, Zhejiang, China

**Keywords:** hyperuricemia, insulin resistance, estimated glucose disposal rate, NHANES, population-based study

## Abstract

**Purpose:**

The estimated glucose disposal rate (eGDR) is a simple and noninvasive clinical measure used to assess insulin resistance (IR), yet its potential utility as a marker for hyperuricemia risk had not been systematically evaluated. This study aimed to investigate the relationship between eGDR and hyperuricemia risk among American adults.

**Methods:**

Data for this cross-sectional study were obtained from the 2007–2018 National Health and Nutrition Examination Survey (NHANES). Hyperuricemia was identified as a serum urate (SU) concentration of ≥7 mg/dL in males and ≥6 mg/dL in females. The relationship between eGDR and hyperuricemia risk was assessed using multivariate logistic regression and restricted cubic spline (RCS) methods, with additional subgroup and interaction analyses performed.

**Results:**

With increasing eGDR values, the prevalence of hyperuricemia decreased significantly (29.93% vs. 19.11% vs. 13.20% vs. 5.03%, ***P*** <0.001). Multivariate logistic regression indicated that eGDR was independently associated with the risk of hyperuricemia after controlling for covariates including demographic, lifestyle, and clinical factors (OR=0.93, 95%CI: 0.90-0.96, ***P*** <0.001). RCS analysis further revealed a nonlinear relationship, with a turning point at eGDR 7.96 mg/kg/min. Subgroup analysis revealed a stronger inverse association between eGDR and hyperuricemia risk in females.

**Conclusions:**

The eGDR is inversely associated with hyperuricemia and appears to be a promising epidemiological tool for evaluating the impact of IR on the risk of hyperuricemia.

## 1 Introduction

Hyperuricemia, characterized by abnormally high uric acid levels in the blood, is a common chronic metabolic condition (1). It serves as a key factor in the development of gout (a very painful long-term systemic inflammatory arthritis caused by the deposition of monosodium urate crystal) (2, 3) and has been increasingly associated with conditions such as diabetes, metabolic syndrome, cardiovascular diseases, and higher mortality rates (4–6). In recent years, the global rise in hyperuricemia cases has placed a considerable burden on healthcare systems and economies (7).

Insulin resistance (IR) is an important pathophysiological risk factor for hyperuricemia (8). IR, with consequent compensatory hyperinsulinemia, can disrupt uric acid homeostasis by altering renal urate excretion and potentially increasing *de novo* uric acid production (8, 9). The hyperinsulinemic-euglycemic clamp remains the most reliable method for measuring insulin resistance; however, its use in large-scale epidemiological studies is constrained by the complexity and time requirements of the procedure (10). The estimated glucose disposal rate (eGDR) is a clinical parameter-based index for evaluating insulin sensitivity (11). Initially developed for type 1 diabetes (T1DM) patients, it incorporates variables such as waist circumference (WC), glycated hemoglobin (HbA1c), and hypertension status (12, 13). Moreover, the recognition exists that these individual risk factors (including central obesity, hypertension, and inflammatory states), integral to the eGDR and often co-manifesting, are capable of mechanistically altering the intricate dynamics between glucose regulation and uric acid levels by exacerbating overall metabolic dysregulation. Lower eGDR values indicate poorer insulin sensitivity and greater IR. Compared with traditional methods such as the homeostasis model assessment of insulin resistance (HOMA-IR) and the triglyceride-glucose (TyG) index, eGDR demonstrates superior performance, is simpler to use, does not require fasting blood samples, and is particularly well-suited for large-scale studies (14, 15). Recently, research has shown that eGDR effectively reflects IR and is strongly linked to metabolic syndrome, cardiovascular diseases, and diabetes complications (11, 16–19).

Although IR is a well-established correlate of hyperuricemia with multiple established measurement indices, a notable research gap persists regarding the eGDR. The potential value of eGDR as a simple, non-fasting metric requiring only basic clinical parameters-which could serve as a robust insulin sensitivity marker particularly advantageous for large-scale epidemiological studies and hyperuricemia risk stratification in diverse populations-remains insufficiently investigated. Given the absence of studies on eGDR and hyperuricemia risk, our research, utilizing the National Health and Nutrition Examination Survey (NHANES) data, examine this relationship in the U.S. population. We predict that increased eGDR values are associated with a reduced risk of hyperuricemia.

## 2 Materials and methods

### 2.1 Study population

Data for this study were drawn from NHANES, a survey conducted by the National Center for Health Statistics at the Centers for Disease Control and Prevention (CDC). The survey used a stratified, randomized, multi-stage sampling approach to ensure a nationally representative sample. Participants underwent physical examinations, completed health and nutrition surveys, and participated in laboratory tests. The NHANES protocol was reviewed and approved by the Ethics Review Board of the National Center for Health Statistics (NCHS), and written informed consent was collected from all participants. Detailed methodologies and datasets are available at https://wwwn.cdc.gov/nchs/nhanes/. The NHANES cycles from 2007 to 2018, comprising 59842 participants, were utilized in this study, with exclusions applied to individuals under 20, pregnant women, and those lacking complete eGDR and uric acid data, resulting in 29328 participants.

### 2.2 Definition of eGDR and hyperuricemia

The eGDR (mg/kg/min) is estimated using the formula: eGDR = 21.158 − (0.09 × WC) − (3.407 × HTN) − (0.551 × HbA1c) (13, 20). In this equation, WC represents waist circumference in centimeters, HTN indicates hypertension status (1 = yes, 0 = no), and HbA1c refers to glycated hemoglobin (%). Hyperuricemia is determined by serum urate (SU) levels of 7 mg/dL or more in men and 6 mg/dL or more in women (21).

### 2.3 Assessment of covariates

In this study, covariates included demographic characteristics (age, gender, and race), socio-economic factors (marital status, income, and education), smoking history, alcohol consumption, diuretics use, health conditions (hypertension, diabetes, cardiovascular disease, chronic kidney disease, and gout), and other indicators such as body mass index (BMI), WC, HbA1c, triglycerides (TG), total cholesterol (TC), high-density lipoprotein cholesterol (HDL-c), and low-density lipoprotein cholesterol (LDL-c). Smoking history encompasses both current and former smoking. Alcohol consumption was determined having consumed at least 12 alcoholic drinks in the past year. Use of diuretics was determined based on responses to the question: “During the past 30 days, have you used or taken any prescription medications?”. Diagnosis of chronic kidney disease was determined by an estimated glomerular filtration rate (eGFR) below 60 mL/min/1.73 m² and/or a urine albumin-to-creatinine ratio (UACR) of 30 mg/g or more. The eGFR was calculated using the Chronic Kidney Disease Epidemiology Collaboration (CKD-EPI) equation, which incorporates age, gender, race, and serum creatinine (Scr) levels (22). Diabetes was diagnosed based on a self-reported history, fasting plasma glucose (FPG) levels of ≥7.0 mmol/L, HbA1c levels of ≥6.5%, or the use of antidiabetic drugs. Hypertension was defined as a self-reported history, systolic blood pressure (SBP) ≥140 mmHg, diastolic blood pressure (DBP) ≥90 mmHg, or the use of antihypertensive medications. Cardiovascular diseases were identified through participants’ self-reported histories of heart attacks, strokes, heart failure, coronary artery disease, or angina. The presence of gout was established through the question: “Has a doctor or other health professional ever told you that you have gout?”. Full methodological details for each variable analyzed in this research are publicly accessible via the NHANES database (https://wwwn.cdc.gov/nchs/nhanes/).

### 2.4 Statistical analysis

In accordance with CDC guidelines, statistical analyses utilized a complex multistage cluster survey design and incorporated sampling weights. Continuous variables were presented as means with 95% confidence intervals (CIs), while categorical variables were summarized as percentages with 95% CIs. Weighted Student’s t-tests and chi-squared tests were used to evaluate group differences in continuous and categorical variables, respectively. Logistic and linear regression models were applied to investigate the relationships between eGDR and hyperuricemia or SU levels. To assess potential nonlinear associations between eGDR and hyperuricemia risk, restricted cubic spline (RCS) regression with four knots was performed, with the median value as the reference point. A two-piecewise regression model was employed to identify intervals, and the Log-likelihood ratio test was used to evaluate the presence of a threshold effect. Subgroup analyses were carried out based on covariate stratification, with the other covariates being adjusted for. Receiver operating characteristic (ROC) curve analysis and decision curve analysis (DCA) were employed to compare the classification accuracy and clinical utility of eGDR with those of other alternative indicators. Statistical analyses in this research were performed using Empower software (http://www.empowerstats.com) and R software (http://www.R-project.org), with a two-sided ***P*** value < 0.05 considered statistically significant.

## 3 Results

### 3.1 Baseline characteristics of study population.

The study population consisted of 29328 participants with a mean age of 47.49 years. The racial composition included 8.64% Mexican Americans, 10.53% Non-Hispanic Blacks, 66.94% Non-Hispanic Whites, 5.90% Other Hispanics, and 7.98% from other racial groups. A weighted analysis was performed to evaluate the general and clinical characteristics of participants with and without hyperuricemia (**Table 1**). The results showed that individuals with hyperuricemia were generally older, predominantly male, more likely to smoke and consume alcohol, and more frequently used diuretics (***P*** <0.01). They also had higher prevalence rates of diabetes, hypertension, chronic kidney disease, cardiovascular disease, gout, as well as elevated BMI, WC, HbA1c, TG, TC, and LDL-c levels (***P*** <0.001). Additionally, they were found to have lower educational attainment and reduced HDL-c levels (***P*** <0.01). Furthermore, eGDR levels were significantly reduced in the hyperuricemia group compared to the non-hyperuricemia group (***P*** <0.001).

**Table 1:** Baseline characteristics of study population, weighted.

Characteristics	Overall (n=29328)	Non-hyperuricemia (n=24359)	Hyperuricemia (n=4969)	*P* value
Age (years)	47.49 (47.04, 47.94)	46.93 (46.46, 47.41)	50.39 (49.75, 51.02)	<0.001
Gender				<0.001
Female	50.90 (50.28, 51.51)	54.65 (53.98, 55.33)	31.18 (29.56, 32.85)	
Male	49.10 (48.49, 49.72)	45.35 (44.67, 46.02)	68.82 (67.15, 70.44)	
Race (%)				<0.001
Mexican American	8.64 (7.25, 10.28)	9.00 (7.56, 10.68)	6.77 (5.45, 8.38)	
Non-Hispanic Black	10.53 (9.22, 12.01)	10.21 (8.96, 11.62)	12.21 (10.43, 14.25)	
Non-Hispanic White	66.94 (64.10, 69.66)	66.64 (63.77, 69.39)	68.51 (65.37, 71.48)	
Other Hispanic	5.90 (5.00, 6.96)	6.18 (5.22, 7.29)	4.48 (3.72, 5.38)	
Other Races	7.98 (7.15, 8.90)	7.97 (7.12, 8.91)	8.04 (6.95, 9.28)	
PIR (%)				0.193
<=1.3	21.62 (20.32, 22.96)	21.77 (20.41, 23.19)	20.81 (19.36, 22.33)	
>1.3, <=3.5	35.41 (34.11, 36.73)	35.14 (33.76, 36.55)	36.83 (34.87, 38.83)	
>3.5	42.98 (40.96, 45.02)	43.09 (41.00, 45.21)	42.36 (39.71, 45.06)	
Education level (above high school) (%)	61.45 (59.62, 63.24)	61.94 (60.06, 63.78)	58.88 (56.32, 61.39)	0.006
Smoking history (%)	44.47 (43.28, 45.67)	43.63 (42.30, 44.97)	48.88 (46.96, 50.80)	<0.001
Alcohol consumption (%)	80.56 (79.38, 81.68)	80.17 (78.92, 81.36)	82.59 (81.00, 84.07)	0.002
Diabetes (%)	12.79 (12.24, 13.36)	11.66 (11.08, 12.26)	18.72 (17.35, 20.18)	<0.001
Hypertension (%)	36.74 (35.71, 37.77)	33.07 (32.00, 34.16)	56.00 (54.12, 57.86)	<0.001
Chronic kidney disease (%)	13.81 (13.21, 14.43)	11.45 (10.89, 12.03)	26.29 (24.60, 28.04)	<0.001
Cardiovascular disease (%)	8.27 (7.81, 8.76)	7.30 (6.82, 7.81)	13.38 (11.98, 14.90)	<0.001
Gout (%)	3.95 (3.63, 4.31)	2.73 (2.45, 3.04)	10.38 (9.24, 11.65)	<0.001
Diuretics (%)	6.94 (6.52, 7.38)	5.12 (4.76, 5.50)	16.51 (15.23, 17.87)	<0.001
BMI (kg/m^2^)	29.00 (28.84, 29.17)	28.38 (28.21, 28.55)	32.31 (31.99, 32.62)	<0.001
WC (cm)	99.34 (98.90, 99.78)	97.53 (97.08, 97.97)	108.85 (108.08, 109.62)	<0.001
HbA1c (%)	5.64 (5.62, 5.65)	5.61 (5.59, 5.63)	5.76 (5.72, 5.79)	<0.001
TG (mmol/L)	1.40 (1.37, 1.43)	1.32 (1.29, 1.35)	1.78 (1.71, 1.86)	<0.001
TC (mmol/L)	4.99 (4.97, 5.02)	4.97 (4.95, 5.00)	5.11 (5.06, 5.16)	<0.001
LDL-c (mmol/L)	2.94 (2.92, 2.97)	2.93 (2.91, 2.95)	3.02 (2.96, 3.07)	0.003
HDL-c (mmol/L)	1.38 (1.37, 1.39)	1.41 (1.39, 1.42)	1.24 (1.22, 1.25)	<0.001
eGDR (mg/kg/min)	7.86 (7.79, 7.93)	8.16 (8.09, 8.24)	6.28 (6.17, 6.40)	<0.001

Weighted analyses to evaluate the general and clinical characteristics of participants with and without hyperuricemia.

PIR, poverty income ratio; BMI, body mass index; WC, waist circumference; HbA1c, glycated hemoglobin; TG, triglycerides; TC, total cholesterol; LDL-c, low-density lipoprotein cholesterol; HDL-c, high-density lipoprotein cholesterol; eGDR, estimated glucose disposal rate.

### 3.2 Baseline characteristics of four different quartiles (1-4) based on increasing eGDR values.

Participants were classified into four groups based on eGDR quartiles (**Table 2**). Compared to those in the lowest quartile, individuals in the higher quartiles were younger, more likely to be female and drinkers, and had lower rates of smoking, diuretic use, diabetes, hypertension, chronic kidney disease, cardiovascular disease, and gout (***P*** <0.001). They also tended to have higher levels of education and a greater PIR (poverty income ratio) (***P*** <0.001). Significant reductions were noted in BMI, WC, HbA1c, TG, TC, and LDL-c levels, while HDL-c levels were significantly higher (***P*** <0.001). Race distribution also differed significantly (***P*** <0.001). SU levels and hyperuricemia prevalence decreased with rising eGDR levels which is in agreement with the previous report (23) (***P*** <0.001).

**Table 2:** Baseline characteristics of four eGDR quartiles (increasing order, 1-4), weighted.

Characteristics	Quartile 1	Quartile 2	Quartile 3	Quartile 4	*P* value
Age (years)	56.73 (56.25, 57.21)	53.20 (52.57, 53.83)	45.06 (44.51, 45.62)	37.70 (37.11, 38.28)	<0.001
Gender					<0.001
Female	44.88 (43.24, 46.53)	51.93 (50.47, 53.38)	44.39 (42.95, 45.84)	61.07 (59.59, 62.52)	
Male	55.12 (53.47, 56.76)	48.07 (46.62, 49.53)	55.61 (54.16, 57.05)	38.93 (37.48, 40.41)	
Race (%)					<0.001
Mexican American	7.13 (5.70, 8.89)	7.49 (6.12, 9.12)	11.87 (9.91, 14.15)	7.70 (6.54, 9.05)	
Non-Hispanic Black	14.01 (11.92, 16.39)	11.44 (9.96, 13.11)	8.35 (7.23, 9.64)	9.12 (7.99, 10.40)	
Non-Hispanic White	68.99 (65.55, 72.24)	68.15 (65.10, 71.06)	65.84 (62.68, 68.88)	65.35 (62.52, 68.07)	
Other Hispanic	4.56 (3.67, 5.65)	5.22 (4.38, 6.22)	6.82 (5.73, 8.09)	6.67 (5.56, 7.98)	
Other Races	5.32 (4.59, 6.15)	7.70 (6.64, 8.91)	7.12 (6.14, 8.24)	11.16 (9.82, 12.64)	
Married (%)	58.82 (56.92, 60.70)	56.96 (55.18, 58.73)	59.01 (57.03, 60.96)	48.60 (46.49, 50.72)	<0.001
PIR (%)					<0.001
<=1.3	22.34 (20.55, 24.25)	20.74 (19.30, 22.26)	21.61 (19.96, 23.37)	21.78 (19.93, 23.75)	
>1.3, <=3.5	37.55 (35.91, 39.23)	37.81 (35.84, 39.82)	34.45 (32.48, 36.48)	32.62 (30.71, 34.58)	
>3.5	40.10 (37.61, 42.65)	41.45 (38.97, 43.97)	43.94 (41.11, 46.80)	45.60 (42.87, 48.36)	
Education level (above high school) (%)	55.69 (53.67, 57.70)	59.29 (56.92, 61.62)	60.13 (57.67, 62.54)	69.10 (66.78, 71.32)	<0.001
Smoking history (%)	51.91 (50.27, 53.54)	47.27 (45.42, 49.13)	43.35 (41.80, 44.92)	37.31 (35.36, 39.29)	<0.001
Alcohol consumption (%)	78.22 (76.64, 79.72)	78.89 (77.45, 80.27)	82.36 (80.70, 83.90)	82.19 (80.59, 83.69)	<0.001
Diabetes (%)	37.17 (35.68, 38.70)	12.63 (11.58, 13.76)	5.20 (4.64, 5.81)	0.95 (0.70, 1.29)	<0.001
Hypertension (%)	95.13 (94.42, 95.76)	64.70 (63.08, 66.29)	2.48 (2.07, 2.97)	0.00 (0.00, 0.00)	<0.001
Chronic kidney disease (%)	27.47 (26.20, 28.79)	17.59 (16.45, 18.78)	7.33 (6.64, 8.10)	6.11 (5.46, 6.84)	<0.001
Cardiovascular disease (%)	18.70 (17.48, 19.99)	11.42 (10.45, 12.47)	3.67 (3.14, 4.30)	1.80 (1.45, 2.24)	<0.001
Gout (%)	9.74 (8.86, 10.70)	4.61 (3.95, 5.39)	2.22 (1.77, 2.78)	0.50 (0.36, 0.68)	<0.001
Diuretics (%)	19.79 (18.56, 21.09)	9.05 (8.14, 10.05)	1.19 (0.94,1.51)	0.53 (0.34, 0.84)	<0.001
BMI (kg/m^2^)	35.43 (35.17, 35.69)	29.84 (29.62, 30.06)	29.05 (28.91, 29.19)	23.20 (23.10, 23.29)	<0.001
WC (cm)	117.09 (116.60, 117.57)	102.09 (101.58, 102.60)	99.94 (99.71, 100.18)	82.39 (82.14, 82.64)	<0.001
HbA1c (%)	6.32 (6.27, 6.36)	5.67 (5.65, 5.70)	5.47 (5.45, 5.48)	5.23 (5.22, 5.24)	<0.001
TG (mmol/L)	1.78 (1.71, 1.85)	1.47 (1.41, 1.52)	1.44 (1.39, 1.48)	0.98 (0.96, 1.00)	<0.001
TC (mmol/L)	4.95 (4.91, 5.00)	5.11 (5.07, 5.16)	5.14 (5.10, 5.18)	4.79 (4.76, 4.82)	<0.001
LDL-c (mmol/L)	2.88 (2.83, 2.92)	3.02 (2.97, 3.08)	3.11 (3.08, 3.15)	2.76 (2.73, 2.80)	<0.001
HDL-c (mmol/L)	1.22 (1.21, 1.23)	1.38 (1.37, 1.40)	1.32 (1.30, 1.33)	1.56 (1.54, 1.58)	<0.001
SU (mg/dL)	6.06 (6.01, 6.12)	5.59 (5.54, 5.64)	5.45 (5.40, 5.50)	4.76 (4.73, 4.80)	<0.001
Hyperuricemia (%)	29.93 (28.53, 31.36)	19.11 (17.81, 20.49)	13.20 (12.07, 14.42)	5.03 (4.41, 5.75)	<0.001

Participants were classified into four quartiles based on increasing eGDR from quartile 1 to quartile 4.

### 3.3 Analyzing the relationship between eGDR and hyperuricemia or SU levels using Logistic and Linear regression analysis.

Our findings demonstrate a significant negative association between elevated eGDR levels and hyperuricemia, which persists across models 1 (OR=0.78, 95%CI: 0.78-0.79, ***P*** <0.001), 2 (OR=0.79, 95%CI: 0.78-0.80, ***P*** <0.001), and 3 (OR=0.93, 95%CI: 0.90-0.96, ***P*** <0.001) (**Table 3**). Further stratification by eGDR quartiles, using the lowest quartile as a reference, shows that individuals in the highest quartile also have a lower risk of hyperuricemia in the fully adjusted model (OR=0.49, 95%CI: 0.38-0.63, ***P*** <0.001). The analysis of SU levels as the dependent variable and eGDR levels as the independent variable through linear regression also demonstrates a negative relationship between them (β=-1.19, 95%CI: -1.98–0.39, ***P*** =0.003) (**Table 4**).

**Table 3:** Logistic regression analysis to assess relation between eGDR and hyperuricemia.

Hyperuricemia	OR (95%CI) *P* value		
	Model 1	Model 2	Model 3
Continuous			
eGDR	0.78 (0.78, 0.79) <0.001	0.79 (0.78, 0.80) <0.001	0.93 (0.90, 0.96) <0.001
Categories			
Q1	reference	reference	reference
Q2	0.53 (0.50, 0.58) <0.001	0.54 (0.50, 0.59) <0.001	1.02 (0.88, 1.18) 0.823
Q3	0.34 (0.31, 0.37) <0.001	0.34 (0.31, 0.38) <0.001	0.75 (0.63, 0.89) 0.001
Q4	0.13 (0.11, 0.14) <0.001	0.13 (0.11, 0.15) <0.001	0.49 (0.38, 0.63) <0.001
*P* for trend	<0.001	<0.001	<0.001

Logistic regression analyses in three different models of adjustment were performed to investigate the relationships between eGDR and hyperuricemia.

OR, odds ratio.

95% CI, 95% confidence interval.

Model 1: non-adjusted.

Model 2: adjusted for age, gender, race, marital status, PIR, education level, smoking, and alcohol consumption.

Model 3: adjusted for age, gender, race, marital status, PIR, education level, smoking, alcohol consumption, diabetes, chronic kidney disease, cardiovascular disease, gout, diuretics, BMI, TG, LDL-c, and HDL-c.

**Table 4:** Linear regression analysis to assess relation between eGDR and SU levels.

SU	β (95%CI) *P* value		
	Model 1	Model 2	Model 3
Continuous			
eGDR	-0.16 (-0.17, -0.16) <0.001	-9.06 (-9.44, -8.69) <0.001	-1.19 (-1.98, -0.39) 0.003
Categories			
Q1	reference	reference	reference
Q2	-0.50 (-0.54, -0.45) <0.001	-25.69 (-28.27, -23.11) <0.001	-1.35 (-5.35, 2.64) 0.506
Q3	-0.64 (-0.68, -0.59) <0.001	-37.50 (-40.21, -34.79) <0.001	-4.92 (-9.31, -0.53) 0.028
Q4	-1.28 (-1.32, -1.24) <0.001	-70.09 (-73.01, -67.17) <0.001	-14.02 (-19.62, -8.42) <0.001
*P* for trend	<0.001	<0.001	<0.001

Linear regression analyses in three different models of adjustment were performed to investigate the relationships between eGDR and SU levels.

95% CI, 95% confidence interval.

Model 1: non-adjusted.

Model 2: adjusted for age, gender, race, marital status, PIR, education level, smoking, and alcohol consumption.

Model 3: adjusted for age, gender, race, marital status, PIR, education level, smoking, alcohol consumption, diabetes, chronic kidney disease, cardiovascular disease, gout, diuretics, BMI, TG, LDL-c, and HDL-c.

### 3.3 RCS analysis

**Figure 1:**
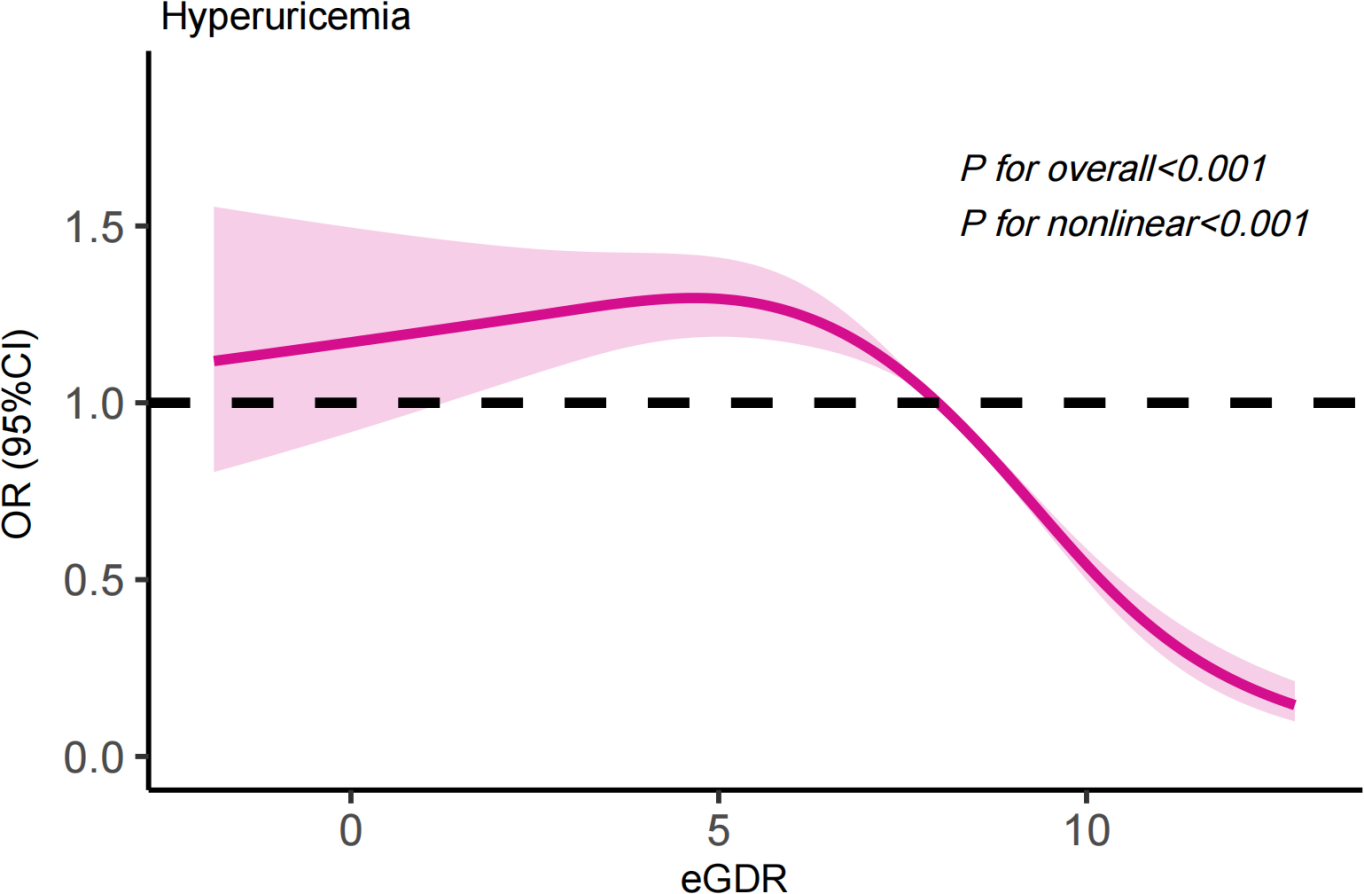
The results of RCS analysis.

RCS analysis to assess non-linearity in the relationship between eGDR and hyperuricemia (**Figure 1**). The threshold effect analysis shows that the inflection point for eGDR levels is 7.66 mg/kg/min, with a more pronounced relationship on the right side (OR=0.76, 95%CI: 0.71-0.82, ***P*** <0.001) compared to the left side (OR=1.02, 95%CI: 0.98-1.06, ***P*** =0.395) (**Table 5**).

**Table 5:** Threshold effect analysis of eGDR on hyperuricemia risk.

Model	OR (95% CI) *P* value
Total	0.93 (0.90, 0.96) <0.001
Breakpoint (K)	7.66
OR1 (<7.96)	1.02 (0.98, 1.06) 0.395
OR2 (>7.96)	0.76 (0.71, 0.82) <0.001
OR2/OR1	0.75 (0.69, 0.82) <0.001
P for logarithmic likelihood ratio	<0.001

OR, odds ratio.

95% CI, 95% confidence interval.

adjusted for age, gender, race, marital status, PIR, education level, smoking, alcohol consumption, diabetes, chronic kidney disease, cardiovascular disease, gout, diuretics, BMI, TG, LDL-c, and HDL-c.

### 3.4 Subgroup analyses

**Figure 2:**
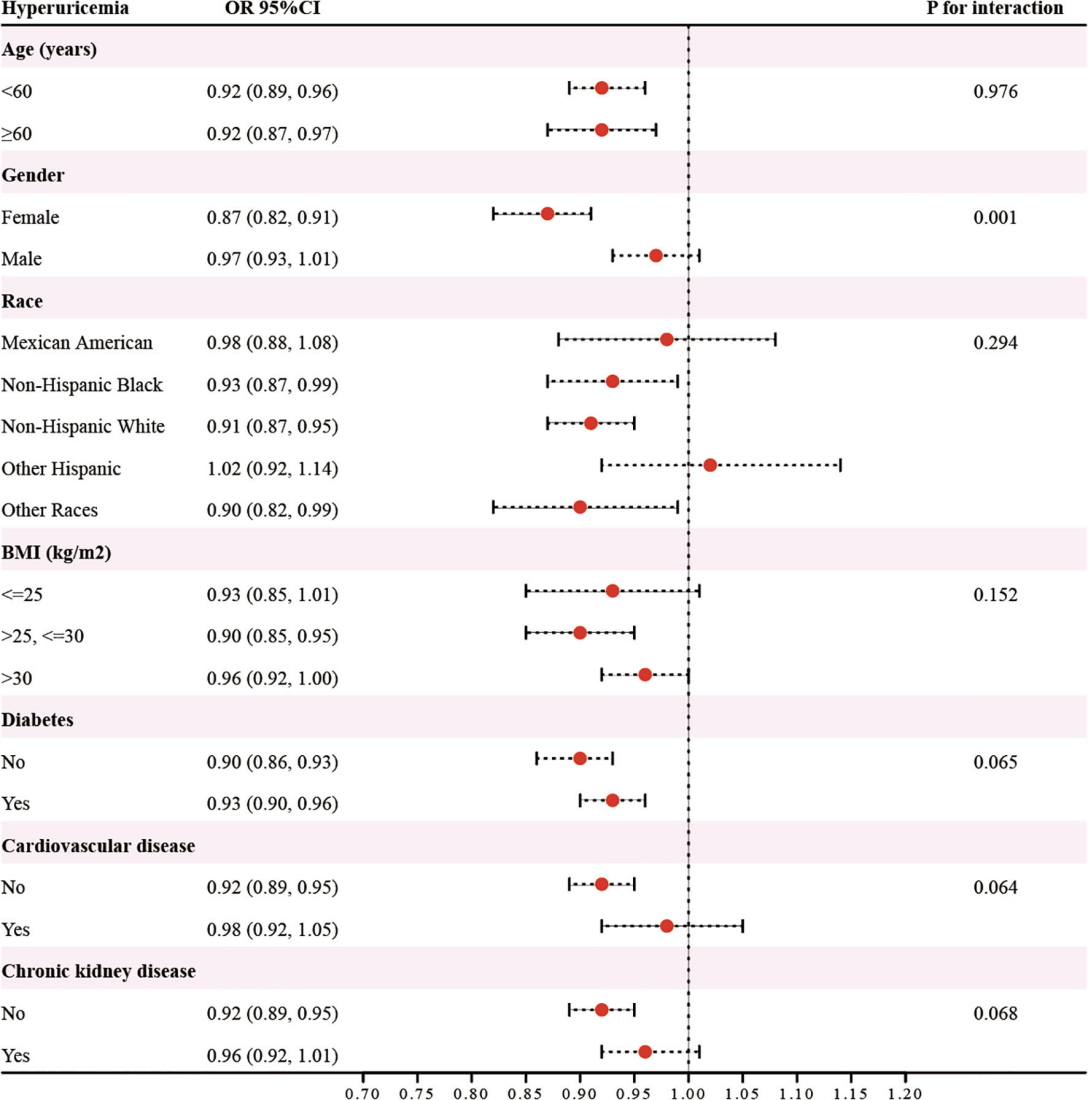
The results of subgroup analysis.

In analyses stratified by variables such as age (<60/≥60 years), gender (female/male), race (Mexican American/Non-Hispanic Black/Non-Hispanic White/Other Hispanic/Other Races), BMI (≤25/25-30/>30 kg/m^2^), diabetes (yes/no), cardiovascular disease (yes/no), and chronic kidney disease (yes/no), the association between eGDR and hyperuricemia risk was significantly stronger in females (OR=0.87, 95%CI: 0.82-0.91) than in males (OR=0.97, 95%CI: 0.93-1.01) (***P*** for interaction=0.001)(**Figure 2**). Across other subgroups, the relationship showed no significant variation (***P*** for interaction > 0.05).

### 3.5 ROC and DCA analyses

**Figure 3:**
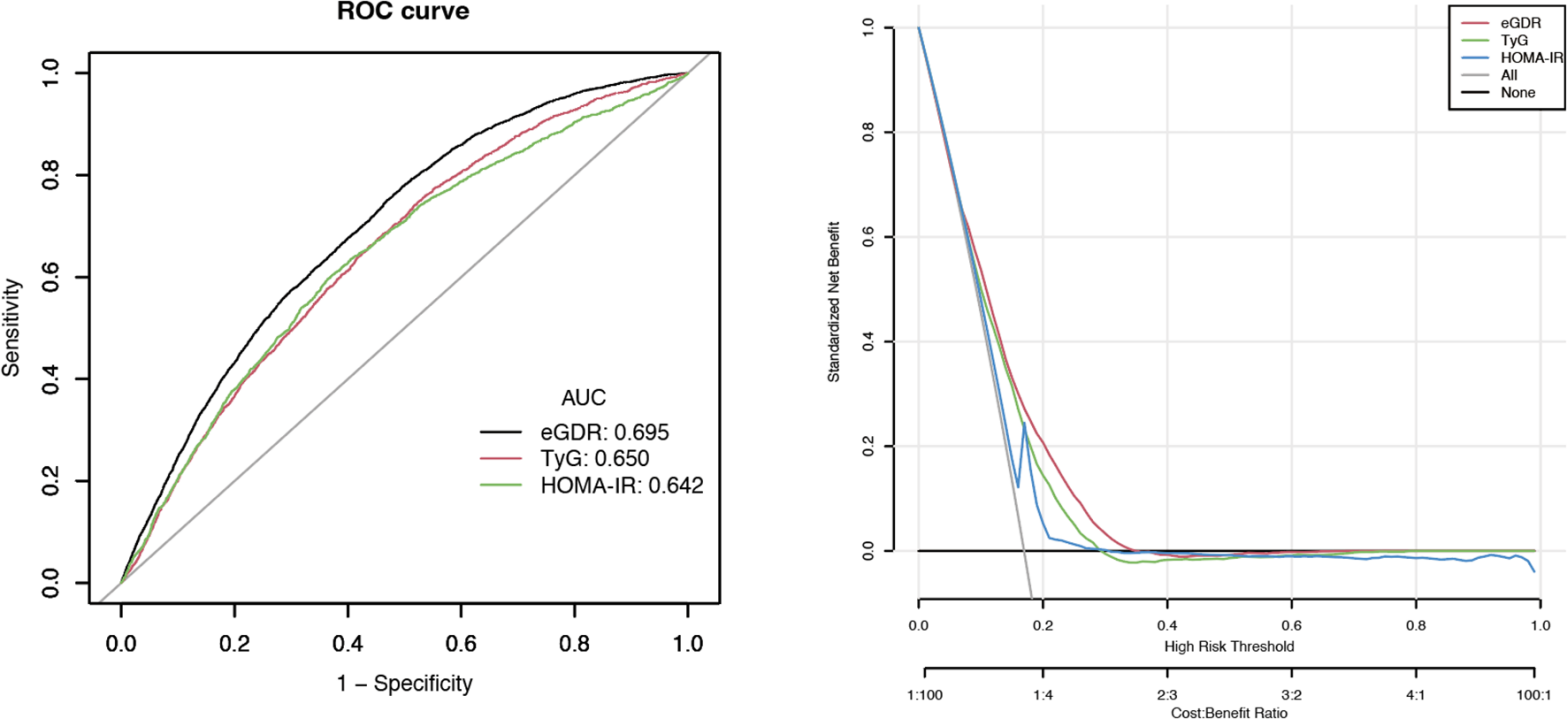
The results of ROC and DCA analyses.

We evaluated eGDR in comparison with other IR surrogates, such as the Triglyceride-Glucose index (TyG) and Homeostasis Model Assessment of Insulin Resistance (HOMA-IR). As illustrated in **Figure 3**, both ROC and DCA analyses were performed. The area under the curves (AUCs) for eGDR, TyG, and HOMA-IR were 69.5%, 65.0%, and 64.2%, respectively, highlighting eGDR as the most effective discriminator for hyperuricemia risk. Moreover, DCA indicated that the eGDR model offered increased net benefit across a broader range of threshold probabilities, reflecting its superior clinical usefulness.

## 4 Discussion

This study reports the results of our investigation about whether the eGDR, used to assess IR, can serve as a straightforward and noninvasive indicator of hyperuricemia. A cross-sectional analysis of 29328 participants revealed a negative and nonlinear correlation between the eGDR and the risk of hyperuricemia.

IR and SU levels were described bidirectionally interconnected because higher SU levels are known to adversely affect the insulin signaling pathway causing IR while IR is a known predictor for the development of hyperuricemia (8, 24). Renal anti-uricosuric effect of insulin was also described preserved in states of IR in human. In compensatory hyperinsulinemia in the state of IR a chronic anti-uricosuric pressure on the kidney cause in hyperuricemia (25). In an *in vitro* experiment, insulin was shown to stimulate urate uptake in human proximal tubular cells (PTC-05) and HEK293T cells and in Xenopus oocyte expression system, where insulin was shown to stimulate urate uptake activity of urate reabsorption transporter, glucose transporter 9 (GLUT9) (26). The eGDR, which is based on clinical parameters, provides a practical and accurate assessment of insulin sensitivity and resistance (27). Specifically, the three components of eGDR reflect IR from different perspectives: Increased WC indicates visceral fat accumulation, which can promote the release of inflammatory factors, exacerbate IR, and reduce renal uric acid excretion, thereby leading to elevated SU levels (28). Hypertension is often associated with IR and may reduce uric acid clearance through renal hemodynamic alterations (29). Elevated HbA1c reflects chronic hyperglycemia and IR, both of which can also influence the renal tubular handling of uric acid (30). Our study found a nonlinear association between eGDR and the risk of hyperuricemia. When eGDR is below the threshold of 7.66, increases in eGDR have limited impact on hyperuricemia risk. However, once eGDR exceeds 7.66, further increases are significantly associated with a reduced risk of hyperuricemia. Therefore, eGDR may serve as a simple and practical screening tool for assessing hyperuricemia risk, especially in primary care settings where more complex measures of IR are unavailable. We propose 7.66 as a potential cutoff value for screening purposes. Our research also revealed that the relationship between eGDR and hyperuricemia risk was stronger in women, potentially reflecting their distinct physiological traits in metabolic regulation (31). Additionally, estrogen plays a role in reducing inflammation and enhancing insulin sensitivity, but its decline after menopause may worsen insulin resistance and disrupt uric acid metabolism (32–35). Estradiol reduces the expression of urate reabsorption transporters, including urate transporter 1 (URAT1) and GLUT9, as well as the efflux transporter ATP-binding cassette sub-family G member 2 (ABCG2), in ovariectomized mice, regardless of hormone replacement therapy (36). Additionally, 17-β-estradiol (E2) has been found to decrease GLUT9 protein levels in human renal tubular epithelial cells (HK2) through estrogen receptor β (ERβ) (37).

The interaction between IR and hyperuricemia is bidirectional, with both conditions sharing metabolic and pathological mechanisms that perpetuate a vicious cycle (4). Obesity, hyperglycemia, and lipid metabolism disorders are common factors linking IR and hyperuricemia, as they promote purine metabolism, oxidative stress, and inflammation, leading to increased uric acid production and decreased insulin sensitivity (38, 39). Clinical evidence showing that allopurinol combined with standard treatment in severe Covid-19 patients reduced oxidative and inflammatory disorders, suggesting that lowering serum urate levels can mitigate oxidative stress (40). In hyperuricemia, reactive oxygen species (ROS) are overproduced during uric acid formation by xanthine oxidases. Both ROS and intracellular uric acid can regulate multiple signaling pathways. For instance, studies demonstrate increased ROS production during 3T3-L1 cell differentiation into adipocytes, indicating that ROS generation correlates with fat accumulation. Interestingly, in fully differentiated 3T3-L1 adipocytes, ROS production was markedly inhibited by NADPH oxidase inhibitors, but not by oxypurinol, rotenone, or thenoyltrifluoroacetone (41).

Uric acid is recognized as an important antioxidant *in vivo*, capable of scavenging ROS such as hydroxyl radicals and peroxynitrite (42, 43). However, under severe oxidative stress, its antioxidant capacity may be overwhelmed, potentially disrupting metabolic homeostasis. Although xanthine oxidase is a key enzyme in uric acid production and a known source of ROS, the relationship between oxidative stress and xanthine oxidase activity remains complex. Some studies indicate that oxidative stress in hyperuricemia may occur independently of xanthine oxidase activity (44), and clinical trials with xanthine oxidase inhibitors (e.g., allopurinol, febuxostat) have yielded inconsistent effects on oxidative stress-related outcomes. Therefore, further research is needed to clarify whether oxidative stress directly disrupts uric acid metabolism or whether their interaction involves additional regulatory mechanisms.

However, this study has limitations. First, given the study’s cross-sectional design, the direction of causality cannot be ascertained, and the role of hyperuricemia in amplifying IR cannot be ruled out. Second, although adjustments were made for several covariates, the effects of unaccounted confounders such as treatment with allopurinol and differences in diuretic use cannot be entirely ruled out. Third, subgroup analyses for factors such as diabetes types, nonalcoholic fatty liver disease (NAFLD) and metabolic syndrome were not performed. Finally, our results, derived from a US population sample, require further verification to ensure their applicability to other demographic groups.

## 5 Conclusion

A nationally representative study among adults aged 20 years or older identified a negative association between the eGDR and the risk of hyperuricemia.

## Data Availability

Publicly available datasets were analyzed in this study. This data can be found here: https://wwwn.cdc.gov/nchs/nhanes.
